# Deep learning-based diffusion tensor image generation model: a proof-of-concept study

**DOI:** 10.1038/s41598-024-53278-8

**Published:** 2024-02-05

**Authors:** Hiroyuki Tatekawa, Daiju Ueda, Hirotaka Takita, Toshimasa Matsumoto, Shannon L. Walston, Yasuhito Mitsuyama, Daisuke Horiuchi, Shu Matsushita, Tatsushi Oura, Yuichiro Tomita, Taro Tsukamoto, Taro Shimono, Yukio Miki

**Affiliations:** Department of Diagnostic and Interventional Radiology, Graduate School of Medicine, Osaka Metropolitan University, 1-4-3, Asahi-Machi, Abeno-Ku, Osaka, 545-8585 Japan

**Keywords:** Neurological disorders, Brain

## Abstract

This study created an image-to-image translation model that synthesizes diffusion tensor images (DTI) from conventional diffusion weighted images, and validated the similarities between the original and synthetic DTI. Thirty-two healthy volunteers were prospectively recruited. DTI and DWI were obtained with six and three directions of the motion probing gradient (MPG), respectively. The identical imaging plane was paired for the image-to-image translation model that synthesized one direction of the MPG from DWI. This process was repeated six times in the respective MPG directions. Regions of interest (ROIs) in the lentiform nucleus, thalamus, posterior limb of the internal capsule, posterior thalamic radiation, and splenium of the corpus callosum were created and applied to maps derived from the original and synthetic DTI. The mean values and signal-to-noise ratio (SNR) of the original and synthetic maps for each ROI were compared. The Bland–Altman plot between the original and synthetic data was evaluated. Although the test dataset showed a larger standard deviation of all values and lower SNR in the synthetic data than in the original data, the Bland–Altman plots showed each plot localizing in a similar distribution. Synthetic DTI could be generated from conventional DWI with an image-to-image translation model.

## Introduction

With the remarkable advancement of artificial intelligence (AI), deep learning technology is being increasingly used in medical imaging, including magnetic resonance imaging (MRI), computed tomography (CT), and positron emission tomography^1^. The image-to-image translation is a popular topic of deep learning^2^. For the central nervous system, several studies have been conducted to generate synthetic images, for example, generating contrast-enhanced MRI of brain tumors from non-contrast-enhanced MRI^3^, generating CT imaging from MRI^4^, and removing motion artifacts from cerebral angiography^5^. These techniques are expected to reduce the examination time, omit minor invasive procedures such as contrast administration, and reduce or prevent radiation exposure.

Previous studies have focused on generating morphological images; however, few studies have been conducted on synthesizing metabolic or physiological images. Recently, methionine PET was developed from contrast-enhanced T1-weighted imaging of subjects with brain tumors^6^. This study estimated the amino acid metabolism of brain tumors and categorized high- and low-grade gliomas. Hence, the image-to-image translation technique could be applied to metabolic images. In addition to PET, metabolic and physiological information can be estimated from MRI using diffusion tensor images (DTI). DTI is an advanced sequence of diffusion weighted images (DWI), requiring equal or more than six directions of the diffusion gradient^7^. DTI has the advantage of being able to predict physiological information, including tissue density, directional strength, continuity of nerve fibers^7^, and brain connectivity^8,9^. However, DTI examinations take longer than conventional DWI. Therefore, the DTI sequence has not been included in routine MR examinations.

If DTI can be obtained as a synthetic image from conventional DWI using an AI technique, detailed physiological information about the brain can be easily assessed within a short inspection time. However, to date, no studies have generated metabolic or physiological MR images, such as DTI, using image-to-image translation techniques. Therefore, this study aimed to create an image-to-image translation AI model that synthesizes DTI from conventional DWI. Further, we aimed to validate the similarities between the original and synthetic DTI.

## Materials and methods

### Subjects

The Ethical Committee of Osaka Metropolitan University Graduate School of Medicine approved this study (IRB:#2021–109), and all participants provided written informed consent before participation. This study complies with the Declaration of Helsinki. Thirty-two healthy volunteers (males, 16; mean age, 30 years; range, 20–38 years) without a history of brain disease or intracranial surgery were prospectively recruited at our institution between July 2021 and January 2022. No subjects were excluded.

### MRI acquisition

MRI was performed using a 3.0-T scanner (Magnetom Vida; Siemens, Erlangen, Germany). The DTI included images with the *b*-value of 0 and 1000 s/mm^2^. Further details are provided in Table 1 and Online Appendix-S1. Three-dimensional T1-magnetization prepared rapid acquisition with gradient-echo images were obtained as anatomical images. Whole-brain DTI with 76 sections were acquired using a 2-dimensional single-shot echo planar imaging sequence with six direction of motion probing gradient (MPG). DTI was denoised and corrected for Gibbs ringing artifacts, motion and eddy currents, susceptibility-induced distortions, and bias field inhomogeneities using the MRtrix3 software (http://www.mrtrix.org/). DWI with three directions of the MPG was also performed using the same parameters and imaging planes. The *b*1000 images of DWI were registered to the corresponding DTI with a 6-degree of freedom rigid transformation using the FLIRT function of the FMRIB Software Library version 6.0 (FSL; Oxford, UK; www.fmrib.ox.ac.uk/fsl) since slight misregistration may have occurred between the DTI and DWI sequences, which affects the precision of the image-to-image translation. In contrast, the *b*0 images of DWI were not registered to the DTI; hence, registered *b*1000 images of DWI and original *b*0 image of DTI were utilized for generating maps of synthetic DTI.

**Table 1:** MRI acquisition parameters.

Parameter	MPRAGE	DTI	DWI
TR (ms)	1800	4500	4500
TE (ms)	2.92	81	81
TI (ms)	900		
FOV (mm)	240 × 240	220 × 220	220 × 220
Imaging matrix	256 × 256	128 × 128	128 × 128
Slice thickness (mm)	0.9	2	2
*b* value (s/mm^2^)		0 and 1000	0 and 1000
MPG directions		6	3
Number of sections		76	76

MPRAGE: T1-magnetization prepared rapid acquisition with gradient echo, DTI: diffusion tensor imaging, DWI: diffusion weighted imaging, TR: repetition time, TE: echo time, TI: inversion time, FOV: field of view, MPG: motion probing gradient.

### Data partition

Thirty-two subjects were randomly divided into training, validation, and test datasets of 26, 2, and 4 subjects, respectively.

### AI model overview

DTI data require equal or more than six MPG directions^7^. Thus, this study synthesized one MPG direction of DTI from DWI (three MPG directions of the x-, y-, and z-axes) and repeated this six times according to each MPG direction. To develop the AI model, DWI (signal intensities of the x-, y-, and z-axis MPG were used as three channels) and one MPG direction of DTI in the same plane (identical signal intensities were input to the three channels) were paired as a png file. Since the order and direction of the MPG were same relative to the gantry for all subjects using the MR machine provided by Siemens, the DTI data could be classified according to the MPG direction. Since signal intensities above 255 on DWI were anticipated to be noise, a signal intensity threshold of 255 was set for DWI to scale the data, to simplify it for the AI model to process. The signal intensities were not normalized since the raw values were important for predicting physiological information.

A deep-learning model was developed based on pix2pix, a generative adversarial network, which is an image-to-image translation model that uses paired images in the training and validation datasets^2^. In this model, the generator adopts a U-Net-based architecture^10^ and the discriminator adopts a convolutional PatchGAN classifier^11^. The model was trained using the training dataset, adjusted to the validation dataset, and evaluated on the test dataset.

**Figure 1:**
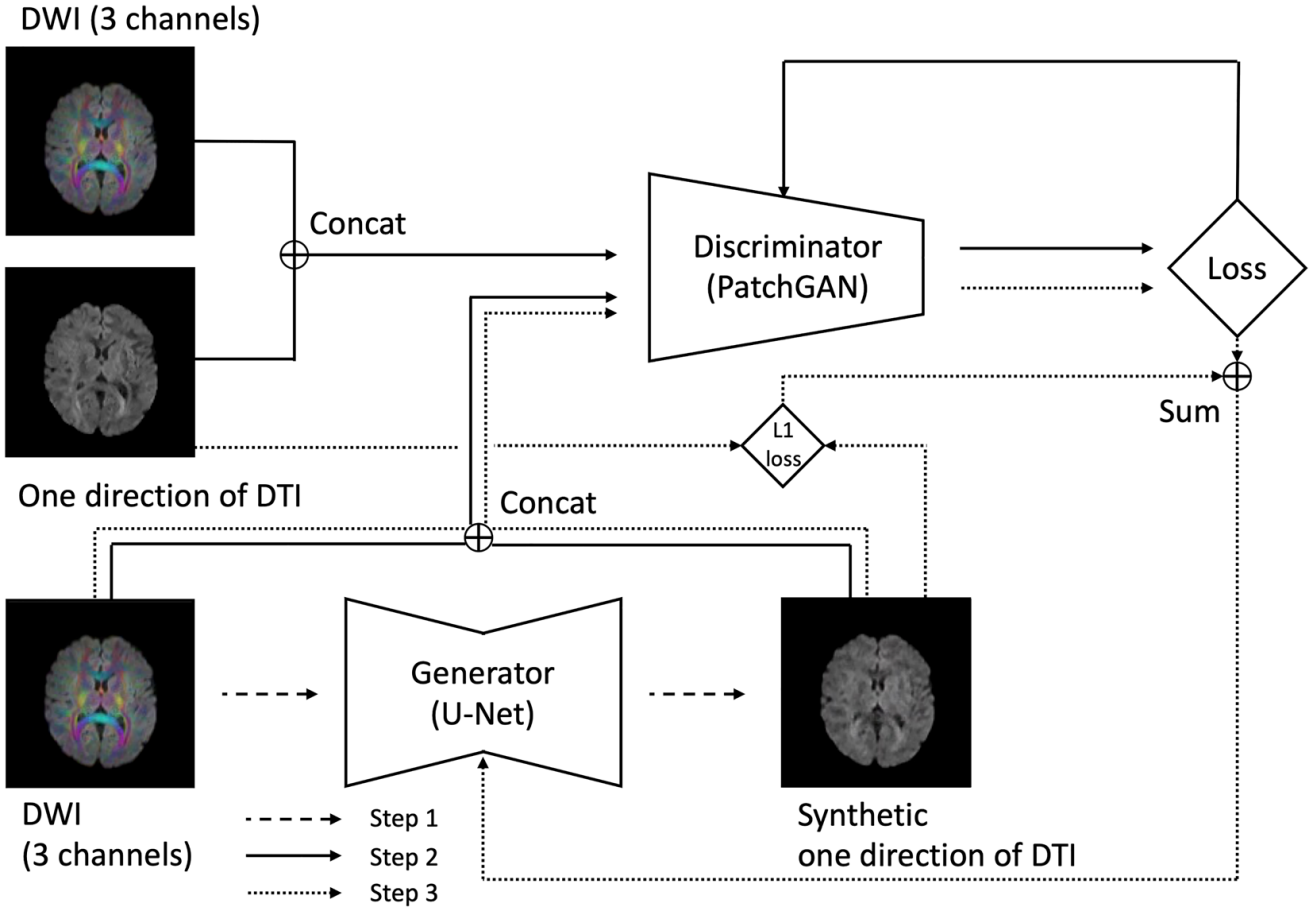
Explanation and overview of the deep learning-based model. Step 1 is the image-generation phase. The generator generates one slice of synthetic DTI of one MPG direction from one slice of DWI (three channels: x-, y-, and z-axis MPG). The original DWI is then concatenated with the synthetic DTI. Step 2 is the learning phase of the discriminator. The concatenated image of one slice of original DWI and synthetic DTI from step 1 or a concatenated image of one slice of original DWI and original DTI from training data are input to the discriminator. The discriminator correctly distinguishes the synthetic and original DTI; therefore, the loss value is set to be small if the discriminator is correct, whereas it is set to be large if the discriminator is wrong. The resulting loss value is backpropagated to the discriminator and the parameters are updated. Step 3 is the learning phase of the generator. The generator generates synthetic DTI with such a high similarity to the original DTI that they can be mistakenly recognized by the discriminator. Hence, the loss value is set to be large if the discriminator is correct, whereas it is set to be small if the discriminator is wrong. In addition, the L1 loss value from the original and synthetic DTI are obtained. These loss values are combined to form the loss value of the generator, and the parameters of the generator are updated. Steps 1 through 3 are repeated as the learning progresses for one MPG direction with the epoch number of 100. DTI: diffusion tensor imaging, MPG: motion probing gradient, DWI: diffusion weighted imaging.

An overview of the AI model is presented in Fig. 1. The learning process was performed six times, because one AI model was needed for one MPG direction. Thus, six AI models were created for each MPG direction. The details are presented in Online Appendices-S2 and S3.

### Visualization of DTI and region of interest (ROI) placement

The separated six MPG directions and 76 slices of the synthetic DTI of the test dataset were 4-dimensionally concatenated as synthetic DTI data. From the original and synthetic DTI, fractional anisotropy (FA), mean diffusivity (MD), axial diffusivity (AD), radial diffusivity (RD), and color-coded maps were generated using the DTIFIT function of FSL software. In this process, the same *b* vector information and *b*0 images were used in the original and synthetic DTI.

**Figure 2:**
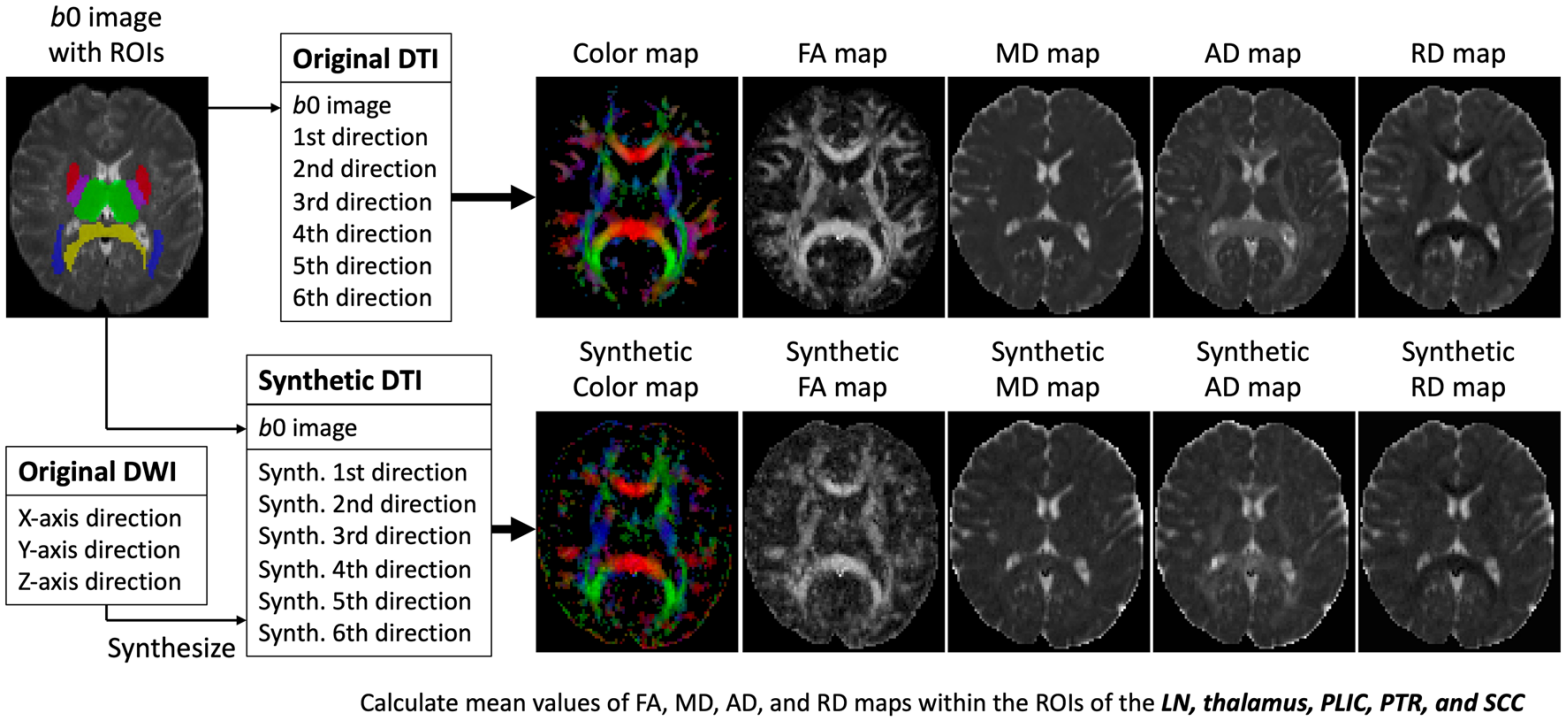
The separated six MPG directions and 76 slices of the synthetic DTI of the test dataset are 4-dimensionally concatenated as one synthetic DTI data. From the original and synthetic DTI, original and synthetic FA map, MD map, AD map, RD map, and color-coded map are generated. ROIs of the LN, thalamus, PLIC, PTR, and SCC are applied to the maps. MPG: motion probing gradient, DTI: diffusion tensor imaging, FA: fractional anisotropy, MD: mean diffusivity, AD: axial diffusivity, RD: radial diffusivity, ROI: region of interest, LN: lentiform nucleus, PLIC: posterior limb of the internal capsule, PTR: posterior thalamic radiation, SCC: splenium of the corpus callosum.

The ROIs of the lentiform nucleus (LN), thalamus, posterior limb of the internal capsule (PLIC), posterior thalamic radiation (PTR), and splenium of the corpus callosum (SCC) were automatically segmented for each subject using the ICBM DTI-81 atlas. Then, the ROIs were applied to the FA, MD, AD, and RD maps derived from the original and synthetic DTI to calculate the mean values and signal-to-noise ratio (SNR) within each ROI (Fig. 2). The ROI creation method is shown in Online Appendix-S4.

### Statistical analyses

The peak signal-to-noise ratio (PSNR) and structural similarity (SSIM) of the separated DTI data were calculated^2,12^. PSNR > 40 indicated a low degree of degradation, whereas SSIM > 0.8 indicated a high degree of similarity^12,13^. For the test dataset, the mean values of the original and synthetic FA/MD/AD/RD maps within each ROI were calculated. The SNR within each ROI was calculated using the following formula: SNR = mean signal intensity/standard deviation (SD). These values were compared between the original and synthetic DTI using a paired-*t* test (*P* < 0.05, indicating statistical significance). A visual inspection of noise quality was conducted by three radiologists, who were blinded to the original and synthetic DTI; they reviewed the color-coded, FA, MD, AD, and RD maps and selected the noisier images for each map. The Bland–Altman plot between the mean values of the FA/MD/AD/RD maps derived from the original and synthetic DTI was evaluated. The analyses were performed using R (version 4.0.0, 2020; R Foundation for Statistical Computing) or GraphPad Prism version 9.4.1 software (GraphPad Software, San Diego, CA, USA; https://www.graphpad.com/scientific-software/prism/).

## Results

The training dataset included 1976 image pairs of one direction of MPG image and DWI from 26 subjects (male, 13; mean age ± SD, 29 ± 5.3 years). The validation dataset included 152 image pairs from two subjects (one male [32 years old] and one female [24 years old]). The test dataset included 304 image pairs from four subjects (male, 2; mean age ± SD, 30 ± 5.8 years).

PSNR and SSIM of the separated DTI data were 32.7 and 0.92, respectively.

The results of the visual inspection for noise quality, as detailed in Online Appendix-S5, indicated that all synthetic DTI maps exhibited more noise compared to their original counterparts.

**Figure 3:**
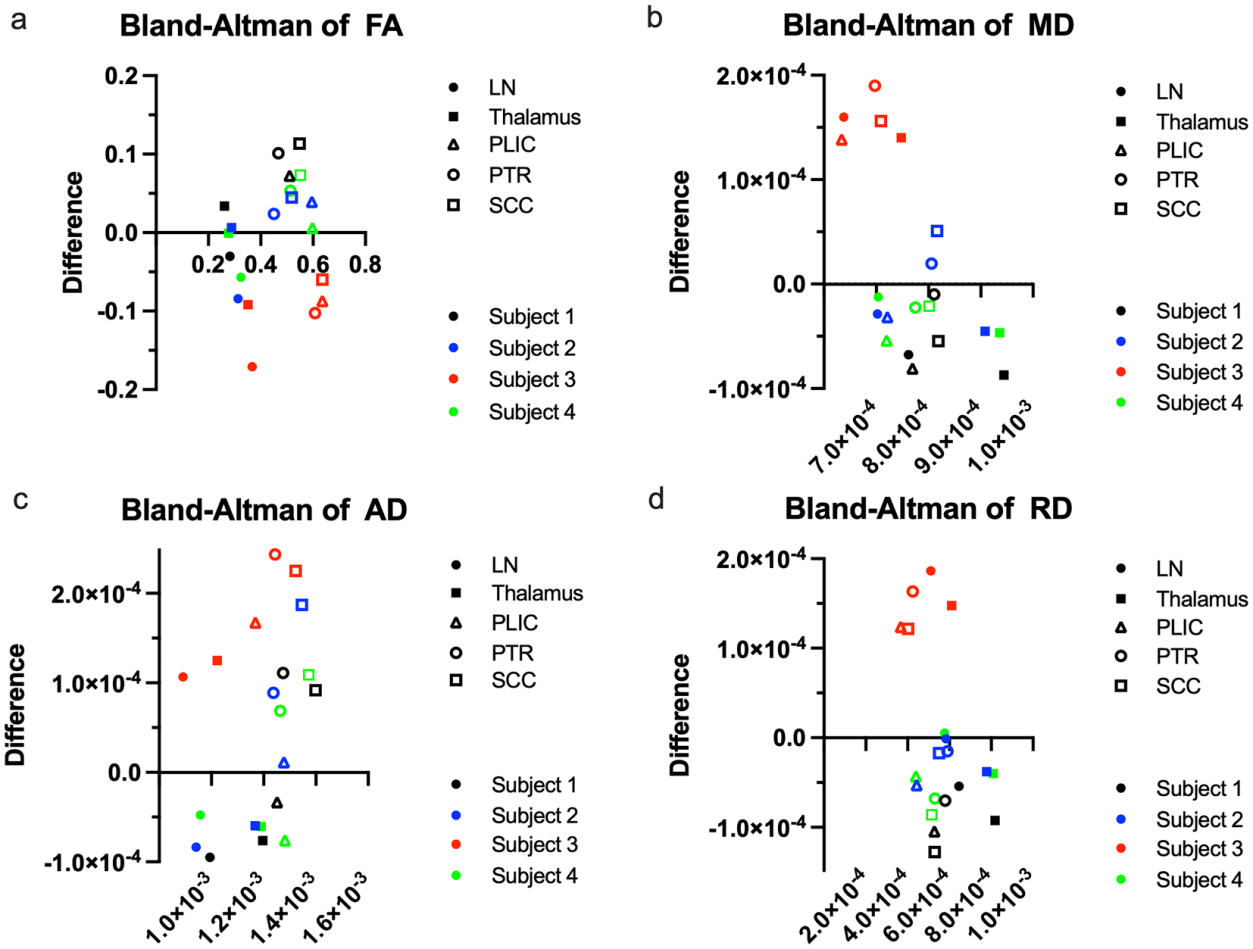
Bland–Altman plots between mean values of the original and synthetic FA/MD/AD/RD maps within each ROI. Except for subject 3, each value shows a similar distribution. In the evaluation of MD and RD, most plots localize under zero, indicating that the synthetic data tend to show higher values than the original data. In the evaluation of AD, plots of the white matter localize above zero, indicating that the synthetic data tend to show lower values than the original data. FA: fractional anisotropy, MD: mean diffusivity, AD: axial diffusivity, RD: radial diffusivity, ROI: region of interest.

Table 2 presents the mean values and SNR of the original and synthetic DTI within each ROI. Only the AD of the PTR and SCC showed significant differences between the original and synthetic maps (*P* = 0.047 and 0.017, respectively), whereas the SD of all analyses was larger in the synthetic maps than in the original maps. The SNR of the FA map within the LN was significantly higher in the synthetic data (*P* = 0.012), whereas the SNR in the other ROIs tended to be lower in the synthetic data than in the original data. Figure 3 shows the Bland–Altman plot of the FA, MD, AD, and RD values within the LN, thalamus, PLIC, PTR, and SCC. Except for the plot corresponding to subject 3, each plot showed a similar distribution. In the evaluation of MD and RD, most values were distributed under zero, indicating that the synthetic MD and RD tended to show higher values than the original data. In the evaluation of AD, values of the white matter were localized above zero, indicating that synthetic AD tended to have lower values than the original data.

**Table 2:** Each value and SNR of original and synthetic maps within the gray and white matter.

		Original DTI	Synthetic DTI	*p* value	SNR of original DTI	SNR of synthetic DTI	*p* value
FA	LN	0.28 ± 0.01	0.36 ± 0.06	0.07	1.76 ± 0.07	2.20 ± 0.10	0.012
	Thalamus	0.28 ± 0.01	0.30 ± 0.07	0.66	2.59 ± 0.08	2.44 ± 0.23	0.26
	PLIC	0.59 ± 0.03	0.58 ± 0.03	0.84	5.09 ± 0.78	4.55 ± 0.09	0.22
	PTR	0.52 ± 0.04	0.50 ± 0.11	0.69	3.75 ± 0.29	3.59 ± 0.65	0.71
	SCC	0.58 ± 0.03	0.54 ± 0.08	0.33	2.51 ± 0.21	2.50 ± 0.19	0.88
MD	LN	7.08 × 10^–4^ ± 0.18 × 10^–4^	6.95 × 10^–4^ ± 1.00 × 10^–4^	0.81	12 ± 2.77	5.16 ± 1.53	0.004
	Thalamus	8.79 × 10^–4^ ± 0.42 × 10^–4^	8.89 × 10^–4^ ± 1.43 × 10^–4^	0.86	2.72 ± 0.82	2.63 ± 0.30	0.77
	PLIC	7.08 × 10^–4^ ± 0.15 × 10^–4^	7.15 × 10^–4^ ± 1.05 × 10^–4^	0.9	20.5 ± 1.35	7.53 ± 1.39	< 0.001
	PTR	7.94 × 10^–4^ ± 0.23 × 10^–4^	7.50 × 10^–4^ ± 0.99 × 10^–4^	0.44	14.66 ± 2.03	7.15 ± 1.05	0.007
	SCC	8.03 × 10^–4^ ± 0.26 × 10^–4^	7.70 × 10^–4^ ± 0.95 × 10^–4^	0.53	4.37 ± 0.62	3.79 ± 0.54	0.17
AD	LN	9.32 × 10^–4^ ± 0.22 × 10^–4^	9.61 × 10^–4^ ± 0.87 × 10^–4^	0.57	5.26 ± 0.36	4.84 ± 0.96	0.48
	Thalamus	1.14 × 10^–3^ ± 0.35 × 10^–4^	1.15 × 10^–3^ ± 1.30 × 10^–4^	0.73	3.08 ± 0.79	2.95 ± 0.38	0.60
	PLIC	1.25 × 10^–3^ ± 0.21 × 10^–4^	1.24 × 10^–3^ ± 1.04 × 10^–4^	0.77	7.83 ± 0.78	6.33 ± 0.86	0.04
	PTR	1.32 × 10^–3^ ± 0.37 × 10^–4^	1.19 × 10^–3^ ± 0.48 × 10-^-4^	0.048	5.57 ± 0.37	5.51 ± 0.76	0.87
	SCC	1.44 × 10^–3^ ± 0.07 × 10^–4^	1.28 × 10^–3^ ± 0.64 × 10^–4^	0.017	4.07 ± 0.39	3.73 ± 0.2	0.19
RD	LN	5.96 × 10^–4^ ± 0.19 × 10^–4^	5.62 × 10^–4^ ± 1.06 × 10^–4^	0.56	6.44 ± 0.72	3.42 ± 1.50	0.016
	Thalamus	7.51 × 10^–4^ ± 0.46 × 10^–4^	7.57 × 10^–4^ ± 1.49 × 10^–4^	0.92	2.33 ± 0.62	4.17 ± 1.98	0.17
	PLIC	4.35 × 10^–4^ ± 0.28 × 10^–4^	4.54 × 10^–4^ ± 1.13 × 10^–4^	0.73	5.96 ± 0.61	2.66 ± 1.09	0.015
	PTR	5.32 × 10^–4^ ± 0.39 × 10^–4^	5.30 × 10^–4^ ± 1.27 × 10^–4^	0.97	6.07 ± 1.09	3.04 ± 1.53	0.038
	SCC	4.86 × 10^–4^ ± 0.38 × 10^–4^	5.13 × 10^–4^ ± 1.16 × 10^–4^	0.65	1.93 ± 0.13	2.88 ± 1.33	0.23

Values indicate mean ± standard deviation.

SNR: signal–noise-ratio, FA: fractional anisotropy, MD: mean diffusivity, AD: axial diffusivity, RD: radial diffusivity, LN: lentiform nucleus, PLIC: posterior limb of capsule, PTR: posterior thalamic radiation, SCC: splenium of corpus callosum.

## Discussion

In this study, DTI was synthesized from conventional DWI using an image-to-image translation model, and the imaging similarity of physiological parameters between the synthetic and original images were evaluated. The variation in all values was larger, and the SNR tended to be lower in synthetic DTI than in original DTI. Except for one subject, the Bland–Altman plots showed that each plot had a similar distribution. The synthetic data of MD and RD showed higher values than the original data, whereas the synthetic data of AD in the white matter showed lower values than the original data.

Synthetic DTI generated using conventional DWI has two main advantages: reduced scan time and reduced motion artifacts. Acquiring synthetic DTI can reduce the scan time compared with acquiring ordinal DTI. Reduced scan time would enable acquiring several MPG directions (64 or more], once the AI model has been established^14^. In fact, using the MR scanner at our institution, the scan times for acquiring DTI with six and 64 MPG directions were 55 s and 5 min 16 s, respectively, whereas that of conventional DWI was shorter compared with DTI. This is particularly beneficial for patients who have difficulty in remaining still during lengthy MR examinations^15^. Shorter scan times can lead to improved imaging quality since there is a decreased likelihood of motion artifacts resulting from movement during the scan.

PSNR and SSIM are often used to evaluate the imaging similarity generated from an image-to-image translation technique. According to previous studies^12,13^, the PSNR and SSIM of synthetic DTI data appear to indicate moderate similarities. Since this study had a small sample size, recruiting more subjects may improve the PSNR and SSIM. However, it is unclear whether the PSNR and SSIM are adequate for evaluating medical imaging, and a gold standard for evaluating physiological imaging has not yet been established. Therefore, a new index for evaluating synthetic medical imaging is required for future studies.

Consistent with the current study, FA is known to be higher in the white matter than in the gray matter, whereas MD shows similar values in the gray and white matter^16^. However, in synthetic DTI, the variation in all values was larger, and the SNR tended to be lower than that of the original DTI. When we reviewed DTI maps visually, the synthetic DTI maps were noisier than the original DTI maps. To further investigate, we conducted the same analysis using denoised and corrected DWI. Nevertheless, the SSIM and PSNR values of the synthetic images remained virtually unchanged (detailed data not shown), indicating that denoising and artifact correction of the input DWI did not notably influence the quality of the synthetic images. Our image-to-image translation model separately learned to generate images in each MPG direction and then concatenated the synthetic images of the six MPG directions. Thus, this model cannot learn how to reduce the noise that occurs during the creation phase of each map. Due to the increased noise, we could not evaluate the differences between synthetic and original DTI using other methods, such as tractography or connectome analyses^17^. Therefore, additional ingenuity may be required to reduce the noise in maps produced by DTI.

For the subject-wise evaluation, the values of one subject (subject 3) seemed to be clustered among the test dataset in the Bland–Altman plot, indicating that the maps of synthetic DTI may have been affected by conventional DWI. Despite thorough visual comparative review of subject 3's original DWI against others, specific causative factors, such as noise or artifacts, remained unidentified. Hence, when using synthetic DTI, a subject-wise coefficient or reference region may be required for imaging harmonization. When we evaluated the distribution of the plots of the MD, AD, and RD values, the synthetic data in the MD and RD maps tended to show higher values and those in the AD maps of the white matter showed lower values than the original data. The number of MPG directions may have affected the map values^18^. A previous study suggested that at least 20 and 30 MPG are necessary for robust estimations of FA and MD, respectively^19^. Since this study focused on a new technique that created synthetic DTI from conventional DWI, a minimum number of MPG directions was selected. Increasing the number of MPG directions may improve the similarities between the values derived from original and synthetic DTI.

This study has several limitations. Firstly, the sample size and the number of MPG directions were small. Increasing the number of participants or obtaining more MPG directions may improve the quality of the synthetic DTI. Secondly, synthetic physiological MR images have not been previously reported; thus, a method for evaluating synthetic medical imaging has not been established. Further validation of the results of this study is required. Conversely, since image-to-image translation techniques have recently been developed and are expected to show further development in various fields, including the medical field, the current results could serve as a reference for future studies. Thirdly, while a 2D GAN model, which utilizes versatile 2D RGB images, was used in this study due to the small dataset, developing a 3D model might enhance the synthesis of DTI and is worth exploring in future research. Models like StarGAN, which can handle multiple domains, may also improve image quality. However, it is expected that the use of a 3D AI model or a specific image generation model would require a larger dataset.

## Conclusion

Although further improvements and validation are required, generating synthetic DTI from conventional DWI using an image-to-image translation model is possible. Overall, our study would promote efficient diagnosis and prevent adverse effects associated with the radiological modalities.

### Supplementary Information

Supplementary Information.

## Data Availability

The datasets used and/or analyzed during the current study are available from the corresponding author on reasonable request.
