# Supplementary material for: Deep learning-based diffusion tensor image generation model: a proof-of-concept study
Source: Sci Rep. 2024 Feb 5;14:2911. doi: 10.1038/s41598-024-53278-8 (PMC10844503; doi:10.1038/s41598-024-53278-8)
Supplement: Supplementary file 1 — Supplementary Information. [file 41598_2024_53278_MOESM1_ESM.pdf]

# Deep Learning-based Diffusion Tensor Image Generation Model: A Proof-of-Concept Study

Hiroyuki Tatekawa[1*], Daiju Ueda[1], Hirotaka Takita[1], Toshimasa Matsumoto[1], Shannon L Walston[1], Yasuhito Mitsuyama[1], Daisuke Horiuchi[1], Shu Matsushita[1], Tatsushi Oura[1], Yuichiro Tomita[1], Taro Tsukamoto[1], Taro Shimono[1], Yukio Miki[1]

[1]Department of Diagnostic and Interventional Radiology, Graduate School of Medicine, Osaka Metropolitan University, Osaka, Japan

**Supplementary Appendix**

**Table of Contents:**

**Supplementary data**

S1) Acquisition parameters of MRI

S2) Deep learning model overview

S3) Machine environment

S4) ROI creation

S5) Visual inspection of noise of maps

**S1) Acquisition parameters of MRI**

All magnetic resonance imaging (MRI) examinations were performed using a 3.0-T scanner with a 32-channel head coil (Magnetom Vida; Siemens, Erlangen, Germany). Three-dimensional T1-magnetization prepared rapid acquisition with gradient echo images (repetition time [TR], 1800 ms; echo time [TE], 2.92 ms; inversion time, 900 ms; field of view [FOV], 240 × 240 mm; imaging matrix, 256 × 256; slice thickness, 0.9 mm without intersection gap) as anatomical images. Whole-brain diffusion tensor imaging (DTI) was performed using a 2-dimensional single-shot echo planar imaging sequence with the following parameters: TR, 4500 ms; TE, 81 ms; *b* value, 1000 s/mm$^2$ with six directions of motion probing gradient (MPG) along with a single *b*0 image (anterior-posterior phase encoding); FOV, 220 × 220 mm; matrix, 128 × 128; and axial imaging plane on the anterior commissure-posterior commissure line. A total of 76 sections (slice thickness: 2 mm without intersection gap) were obtained. The order and directions of the MPG were same relative to the MR gantry in the MR machine provided by Siemens. Before the subsequent procedure, DTI was denoised and corrected for Gibbs ringing artifacts, motion and eddy currents, susceptibility-induced distortions, and bias field inhomogeneities using the MRtrix3 software (http://www.mrtrix.org/). Diffusion-weighted imaging (DWI) in the three directions of the MPG was also performed with the same parameters and imaging planes.

**S2) Deep learning model overview**

A deep learning model was developed based on pix2pix, a generative adversarial network, which is an image-to-image translation model that uses paired images in training and validation datasets [1]. In this model, the generator adopts a U-Net-based architecture [2] and the discriminator adopts a convolutional PatchGAN classifier [3]. The model was trained on the training dataset, adjusted to the validation dataset, and evaluated on a test dataset.

The model-development phase includes 3 steps. Step 1 is the image generation phase. The generator generates one slice of synthetic diffusion tensor images (DTI) in one motion probing gradient (MPG) direction from one slice of diffusion-weighted images (DWI). The original DWI is then concatenated with the synthetic DTI. Step 2 is the learning phase of the discriminator. The concatenated image of one slice of the original DWI and synthetic DTI from step 1, or a concatenated image of one slice of the original DWI and original DTI from the training data are input to the discriminator. The purpose of the discriminator is to correctly distinguish the synthetic and original DTI. Therefore, the loss value is set to be small or large if the discriminator is correct or wrong, respectively. The resulting loss value is backpropagated to the discriminator and the parameters are updated. Step 3 is the learning phase of the generator. The generator generates synthetic DTI with such a high similarity to the original DTI that they can be mistakenly recognized by the discriminator. Hence, the loss value is set to be large if the discriminator is correct, whereas it is set to be small if the discriminator is wrong. In addition, the L1 loss values from the original and synthetic DTI are obtained. These loss values are combined to form the loss value of the generator, and the parameters of the generator are updated. Steps 1–3 are repeated as the learning progresses of one MPG direction, with an epoch number of 100. These processes were performed six times, and six AI models were created for each MPG direction.

The hyperparameters were tuned for the optimizer, learning rate, and batch size. As optimizers, stochastic gradient descent, Adam, and Nadam were evaluated; for the learning rate,

the searching range was 0.001–0.05; for the batch size, the search range was 32–256. Upon completion of the hyperparameter tuning, the final hyperparameters were as follows: (a) optimizer, Adam; (b) learning ratio, 0.0002; (c) momentum parameters, $\beta1 = 0.5$ and $\beta2 = 0.999$; and (d) batch size, 128.

## S3) Machine environment

To create the image-to-image translation model, Ubuntu 20.04 (Canonical, London, England) with the PyTorch [4] deep learning framework was used. The machine contains an Intel i5 3570k 3.4-gHz processor (Intel, Santa Clara, CA), 32 GB of RAM, and a 12 GB Nvidia Titan V graphics processing unit (Nvidia Corporation, Santa Clara, CA).

**S4) Detailed ROI placement**

To obtain representative gray matter regions of interest (ROIs), the ROIs of the lentiform nucleus (LN) and thalamus were segmented from the anatomical images using the FIRST function of the FSL software. To obtain representative white matter ROIs, the ROIs of the posterior limb of the internal capsule (PLIC), posterior thalamic radiation (PTR), and splenium of the corpus callosum (SCC) of ICBM DTI-81 Atlas were selected. The nerve fibers of the PLIC, PTR, and SCC mainly run in the superior-inferior, anterior-posterior, and left-right directions in the brain, respectively. First, the anatomical image was nonlinearly registered to the ICBM T1 Atlas, and the transform and inverse-transform matrices were calculated using the FLIRT and FNIRT functions of the FSL software. Second, the inverse-transform matrices (created in the first step) were applied to the ROIs on the ICBM DTI-81 Atlas to create white matter ROIs in each anatomical space. Third, *b*0 image of the DTI was aligned to the anatomical image, and the transform and inverse-transform matrices were calculated with a 6-degree freedom rigid transformation using the FLIRT function. Finally, inverse-transform matrices (created in the third step) were applied to the representative gray and white matter ROIs in each anatomical space to create ROIs of the LN, thalamus, PLIC, PTR, and SCC in each DTI space. All ROIs were validated by checking whether the registration succeeded on the *b*0 image and color-coded map by a radiologist (H.T., with 15 years of experience).

### S5) Visual inspection of noise of maps

Three radiologists who were blinded to the original and synthetic DTI reviewed the color-coded, FA, MD, AD, and RD maps and selected the noisier images for each map.

The noisier maps were correctly selected by all raters. As shown in the example slice of the maps below, the synthetic maps were noisier than the original maps.

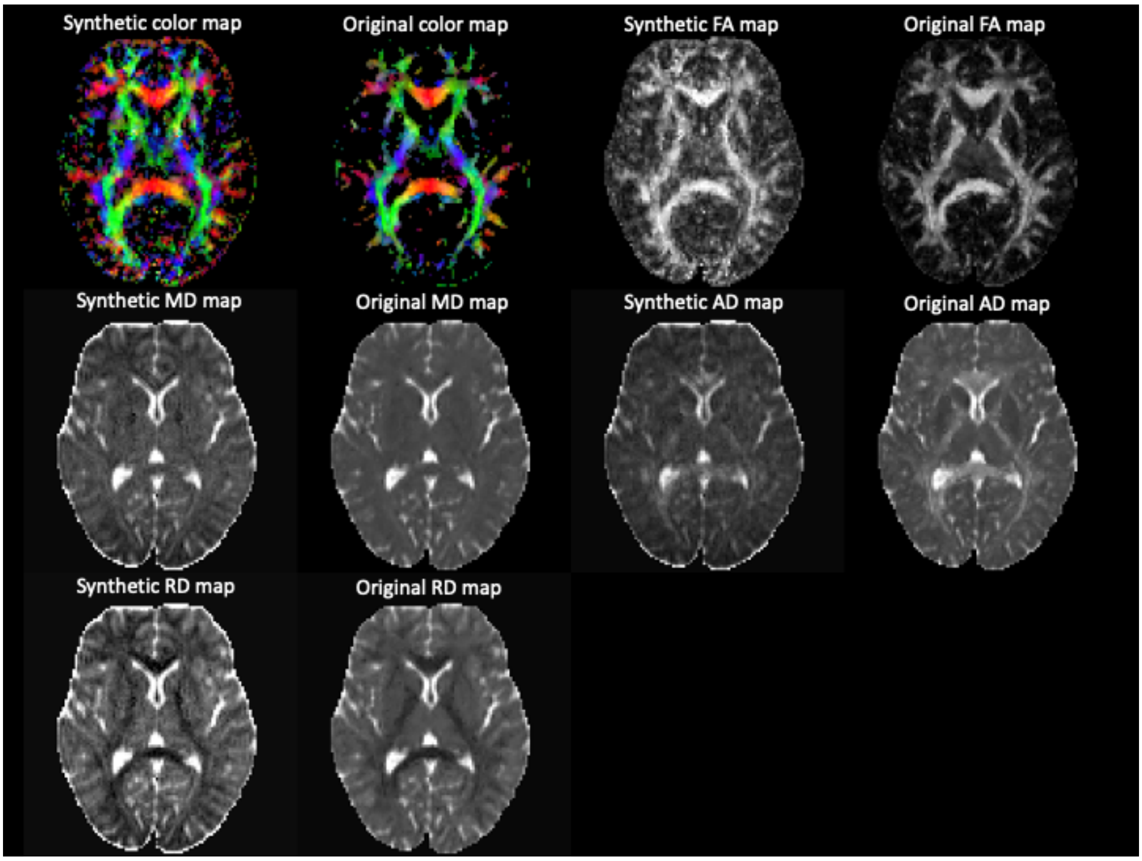

**References**

1. Isola P, Zhu JY, Zhou T, Efros AA (2017) Image-to-image translation with conditional adversarial networks, vol 2017 IEEE Conference on Computer Vision and Pattern Recognition (CVPR), p 5967–5976. https://doi.org/10.1109/CVPR.2017.632

2. Ronneberger O, Fischer P, Brox T (2015) U-net: convolutional networks for biomedical image segmentation. Med Image Comput Comput Assist Interv MICCAI Proceedings, Part III, vol 18: 18th International Conference, Munich, Germany, Oct 5–9, 2015. Springer, p 234–241

3. Li C, Wand M (2016) Precomputed real-time texture synthesis with markovian generative adversarial networks. Comput Vis–ECCV Proceedings, Part III, vol 14: 14th European Conference, Amsterdam, The Netherlands, Oct 11–14, 2016. Springer, p 702–716

4. Paszke A, Gross S, Massa F, Lerer A, Bradbury J, Chanan G, Killeen T, Lin Z, Gimelshein N, Antiga L, Desmaison A, Köpf A, Yang E, DeVito Z, Raison M, Tejani A, Chilamkurthy S, Steiner B, Fang L, Bai J, Chintala S (2019); p PyTorch: an imperative style, high-performance deep learning library. In: Proceedings, 33rd international conference on neural information processing systems: Curran Associates Inc.:article 721
